# Microenvironments created by liquid-liquid phase transition control the dynamic distribution of bacterial division FtsZ protein

**DOI:** 10.1038/srep35140

**Published:** 2016-10-11

**Authors:** Begoña Monterroso, Silvia Zorrilla, Marta Sobrinos-Sanguino, Christine D. Keating, Germán Rivas

**Affiliations:** 1 Centro de Investigaciones Biológicas, Consejo Superior de Investigaciones Científicas (CSIC), 28040, Madrid, Spain; 2 Department of Chemistry, Pennsylvania State University, University Park, Pennsylvania 16802, USA

## Abstract

The influence of membrane-free microcompartments resulting from crowding-induced liquid/liquid phase separation (LLPS) on the dynamic spatial organization of FtsZ, the main component of the bacterial division machinery, has been studied using several LLPS systems. The GTP-dependent assembly cycle of FtsZ is thought to be crucial for the formation of the septal ring, which is highly regulated in time and space. We found that FtsZ accumulates in one of the phases and/or at the interface, depending on the system composition and on the oligomerization state of the protein. These results were observed both in bulk LLPS and in lipid-stabilized, phase-separated aqueous microdroplets. The visualization of the droplets revealed that both the location and structural arrangement of FtsZ filaments is determined by the nature of the LLPS. Relocation upon depolymerization of the dynamic filaments suggests the protein may shift among microenvironments in response to changes in its association state. The existence of these dynamic compartments driven by phase transitions can alter the local composition and reactivity of FtsZ during its life cycle acting as a nonspecific modulating factor of cell function.

Cells are complex entities spatially organized into compartments in which proteins and other metabolites accumulate and perform specialized functions^1^. Although compartmentalization is more obvious in eukaryotic cells, which contain membrane surrounded organelles and nucleus, it is now widely recognized that both eukaryotic and prokaryotic cells are divided in subcompartments^1^,^2^,^3^ thought to be important for many cellular processes. However, *in vitro* studies of their role in, and impact on, these processes remain scarce, particularly for bacterial microenvironments. The bacterial nucleoid is hypothesized to be a liquid phase condensed by a combination of multivalent cations, such as spermine and spermidine, DNA-binding proteins and other crowding agents^4^,^5^,^6^. It is very similar to a complex coacervate, liquid droplets formed by phase separation of mixtures of macromolecules with opposite charges that retain a large amount of solvent^7^. Additionally, highly dynamic membraneless organelles formed by assemblies in direct contact with the surroundings like carboxysomes and magnetosomes in prokaryotes and P granules, stress granules, nucleoli or Cajal bodies in eukaryotes, have been described^1^,^2^,^3^,^8^,^9^,^10^,^11^,^12^. These cellular bodies may be considered liquid droplet phases, suggested to be formed via intracellular phase separation^3^. The differential partition of molecules in these “liquid organelles” might provide additional levels of control for essential biological processes such as bacterial division that need to be tightly regulated in space and time.

The division of *Escherichia coli* is a good example of such orderly processes, described as being initiated by the formation of a dynamic ring in which the FtsZ protein–a GTPase structurally similar to eukaryotic tubulin–localises at midcell together with a few other proteins to assemble the molecular machinery effecting cytokinesis (the divisome)^13^. The Z-ring results from a self-organizing process in which the initial pathway is the GTP-dependent polymerization of FtsZ. Upon addition of GTP, FtsZ forms single-stranded flexible filaments that are shorter than the *E. coli* cell perimeter and continuously exchange subunits with the pool of unassembled protein^14^,^15^. The filaments self-assemble following a cooperative reaction, requiring a critical concentration for assembly. GTP depletion results in FtsZ depolymerization due to accumulation of GDP^14^,^15^ (Fig. 1). When bound to GDP, FtsZ monomers self-associate in a non-cooperative manner to form dimers and short oligomers considerably smaller than the filaments elicited by GTP^16^. Two additional proteins, FtsA and ZipA, are required to attach the FtsZ polymers to the cytoplasmic membrane, forming the first complex of the divisome – the proto-ring. The position of the Z-ring is regulated by two negative control systems that independently inhibit rings from assembling at locations other than midcell: the MinCDE complex and nucleoid occlusion^13^,^17^,^18^. It has been recently proposed that, in addition to these two canonical site-selection systems, the Ter-linkage^19^ participates in Z-ring positioning and that, even in the absence of these three mechanisms, Z-ring assembly mainly occurs next to midcell, suggesting that additional factors that remain to be determined may contribute to Z-ring positioning^20^.

The polymerization of FtsZ takes place inside cells and, hence, in crowded and highly heterogeneous surroundings likely containing microenvironments, which may influence FtsZ assembly and location^21^. Previous studies have shown that the single stranded FtsZ filaments formed in solution in the presence of GTP interact laterally with each other forming higher order structures at high concentrations of unrelated polymers including Ficoll, dextran or PEG as well as certain proteins like ovomucoid^22^,^23^,^24^. These crowders, individually or mixed, also enhance the tendency of the protein to assemble, the extent of the effect being dependent on crowder properties and the composition of the mixture and, in general, a nonadditive behaviour is observed^24^. This strongly suggests that the polymerization of FtsZ is influenced by the high concentration of macromolecules in the bacterial cytoplasm and also by their nature.

Liquid/liquid phase separation (LLPS) is common in solutions having high concentrations of (bio)macromolecules. In the cytoplasm and nucleoplasm of eukaryotic cells, LLPS can provide distinct environments with particular physicochemical properties^1^,^2^,^3^. They originate from the different effects of macromolecules in the two phases on the structure and solvent properties of water^25^, creating distinct environments commonly leading to unequal partition of solutes (small organic molecules, proteins, nucleic acids, RNPs, etc.) which can be enhanced by, but does not generally require, their interactions with phase forming macromolecules^26^,^27^,^28^,^29^. LLPS has been less studied in bacterial cells, which due to their smaller size are difficult to image at high resolution. Recent work by Huck and coworkers demonstrated an advantage to LLPS in bacterial cell lysate, where transcription rates increased upon phase separation in lysate droplets^30^.

Experimental determinations using LLPS systems to mimic cellular microenvironments have shown that the selective accumulation of molecules in one of the phases may deeply influence the location and rates of enzymatic reactions^31^,^32^,^33^,^34^. Larger structures such as lipid vesicles and protein aggregates can collect at the aqueous/aqueous interface, and this interfacial assembly can stabilize droplets against coalescence^35^,^36^,^37^. The interface can further serve as a reservoir for excess lipid membrane: Dimova and coworkers demonstrated that lipid nanotubes pulled from giant liposome membranes were stored at the interface and incorporated back into the bilayer under liposome expansion^38^. Phase separation and membrane accumulation at the aqueous/aqueous phase boundary can also facilitate the separation of LLPS-containing liposomes into distinct daughter vesicles^39^.

This work explores the assembly of FtsZ in the presence of coexisting liquid phases to mimic intracellular compartmentalization. Although macromolecular crowding is known to impact FtsZ polymerization, there have been no studies of this protein in systems that contain LLPS. We selected mixtures of crowders of different nature providing FtsZ with diverse environments and determined the distribution and partition of assembled and unassembled FtsZ in these systems. The potential functional implications of the preferential distribution of FtsZ polymers in microcompartments formed by phase transitions in cell-like crowded solutions are discussed. Prior work on functional consequences of intracellular microenvironments has focused on biomolecule partitioning and hypothesized that (co)localisation increases rates and regulation of enzymatic processes. This work adds a new dimension to thinking about potential intracellular roles for LLPS by showing how microenvironments might influence the distribution of key components of the bacterial cell division system as a function of their polymerization state.

## Results

**Figure 1:**
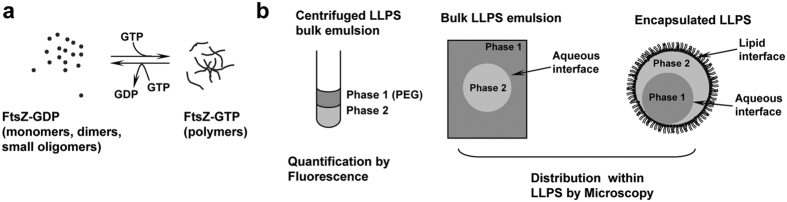
Overview of the biological system and experimental procedure. (**a**) Scheme of the association reactions of FtsZ. When bound to GDP, FtsZ is found as an ensemble of species of small size^16^. Upon GTP binding, and above the critical concentration of polymerization, FtsZ self assembles forming one subunit thick polymers of a defined size^59^,^60^, that in the presence of crowding agents interact laterally forming higher order structures^22^,^23^,^24^. Depletion of the nucleotide by FtsZ GTPase activity increases the ratio of GDP, and the protein depolymerizes. (**b**) Experimental approaches followed. Left, LLPS systems with labelled FtsZ were centrifuged after thoroughly mixing. Both phases separate, the PEG-rich one being always located in the top part of the solution. The fluorescence in each phase is measured allowing quantification of FtsZ concentration. Qualitative distribution and organization of FtsZ was assessed by microscopy using bulk LLPS emulsions (middle) and LLPS emulsions encapsulated in lipid coated microdroplets (right). Each of these approaches was done with the three different LLPS compositions described in the main text.

To determine the effect of LLPS on the distribution of FtsZ species, we analysed the partition of the protein in biphasic systems of three different compositions. As polymerization is crucial for the function of FtsZ, we compared the behaviour of the polymers triggered by GTP with that of the small species the protein forms when bound to GDP. Three experimental approaches were conducted with all LLPS systems tested (Fig. 1). Bulk emulsion and centrifuged bulk emulsion allowed the qualitative characterization of the distribution of the species and its quantification by fluorescence, respectively. Encapsulation of LLPS systems inside microscale lipid droplets was optimized for the characterization of the effect of relevant elements like boundaries and confinement in the distribution of the species and to evaluate the modulation of FtsZ location in response to changes in its association state.

### The distribution of FtsZ in PEG/dextran LLPS systems is inhomogeneous and largely influenced by its association state

**Figure 2:**
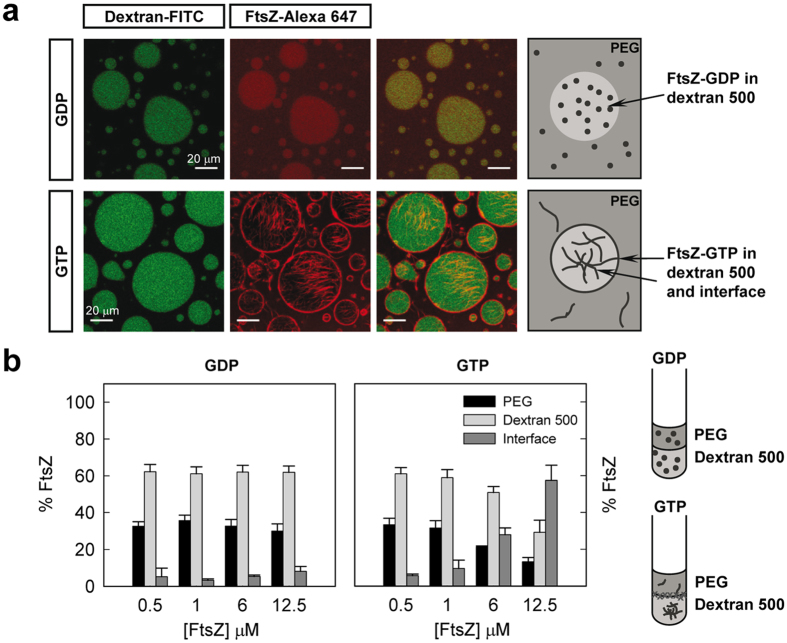
Distribution of FtsZ in the PEG/dextran 500 LLPS system. (**a**) Representative confocal images of FtsZ within the PEG/dextran 500 (3:1) emulsions in the absence and presence of 1 mM GTP. Total FtsZ concentration was 7 μM. A schematic illustration of the disposition of FtsZ within the phases is depicted on the right. (**b**) Concentration dependence of the distribution of FtsZ within the mixture as determined by fluorescence, together with an illustration on the right, in the absence and presence of 1 mM GTP. Data are the average of at least 3 independent measurements ± SD.

**Figure 3:**
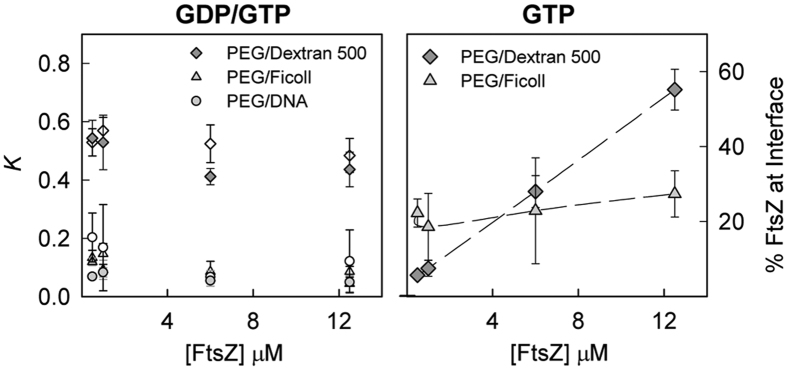
Partition coefficients and interfacial content of FtsZ in several binary mixtures. Left, evolution of the partition coefficient, *K*, with FtsZ concentration in the absence (open) and presence of GTP (solid). Right, variation of the percentage of FtsZ polymers at the interface of the specified LLPS systems with increasing protein concentration. Data are the average of at least 3 independent measurements ± SD.

The first two-phase system selected to probe FtsZ behaviour was PEG/dextran 500, an LLPS system composed of two neutral polymers earlier used in phase separation studies^33^,^40^,^41^. Confocal images of unassembled FtsZ-GDP showed colocalisation between FtsZ-Alexa 647 and fluorescein labelled dextran 500 droplets, indicating that most of the protein localises in this phase (Fig. 2a). Significant FtsZ signal was also detected in the regions enriched in PEG, while no obvious accumulation of the protein was observed at the liquid/liquid interface between the dextran droplets and the PEG rich phase. Fluorescence spectroscopy based determinations of the protein content in each of the phases indicated that ∼60% FtsZ partitioned at the dextran-rich phase, ∼30% into the PEG-rich phase and less than 10% was found at the interface (Fig. 2b). The partition coefficient, *K,* was around 0.5, it did not substantially change within the 0.5–12 μM FtsZ concentration interval (Fig. 3) and, under these conditions, was not sensitive to changes in the relative volumes of the two phases (Supplementary Fig. S1).

To assess the influence of FtsZ polymerization on its distribution in the PEG/dextran 500 system we studied the partition of the protein upon addition of GTP. Colocalisation of FtsZ-Alexa 647 polymers and dextran-FITC droplets was observed in the confocal images of the emulsion, with a remarkable accumulation of protein around the dextran droplets at the interface with the PEG phase (Fig. 2a). Analysis of the distribution of FtsZ-GTP by fluorescence showed a strong dependence on protein concentration (Fig. 2b). Below 1 μM FtsZ the behaviour of FtsZ-GTP was comparable to that observed for FtsZ-GDP, probably because at this low concentration most of the protein remains unassembled and the partition of FtsZ-GDP and FtsZ-GTP species may be similar. Above 1 μM FtsZ the amount of protein in the two phases decreased and an important fraction of the protein located at the interface between the two crowding polymers, in good agreement with the confocal images of the emulsion. The fraction of protein at the interface increased with protein concentration, likely due to an increase in the amount of assembled protein. The fraction of protein at the PEG and dextran 500 phases concomitantly decreased, rendering a nearly constant partition coefficient of 0.4–0.5 similar to that determined for FtsZ-GDP (Fig. 3). The accumulation of the protein polymers at the liquid/liquid interface in the tubes was clear to the naked eye especially at the higher concentrations of FtsZ used (Supplementary Fig. S2).

We conclude that the distribution of FtsZ species in the PEG/dextran 500 system was uneven and largely dictated by its association state. FtsZ species were mainly located in the dextran, rather than in the more hydrophobic PEG phase^25^ and the formation of thick bundles induced by GTP under crowding conditions favoured location of the protein at the interface between these two crowding polymers.

### The LLPS system composition determines the final distribution of FtsZ species

**Figure 4:**
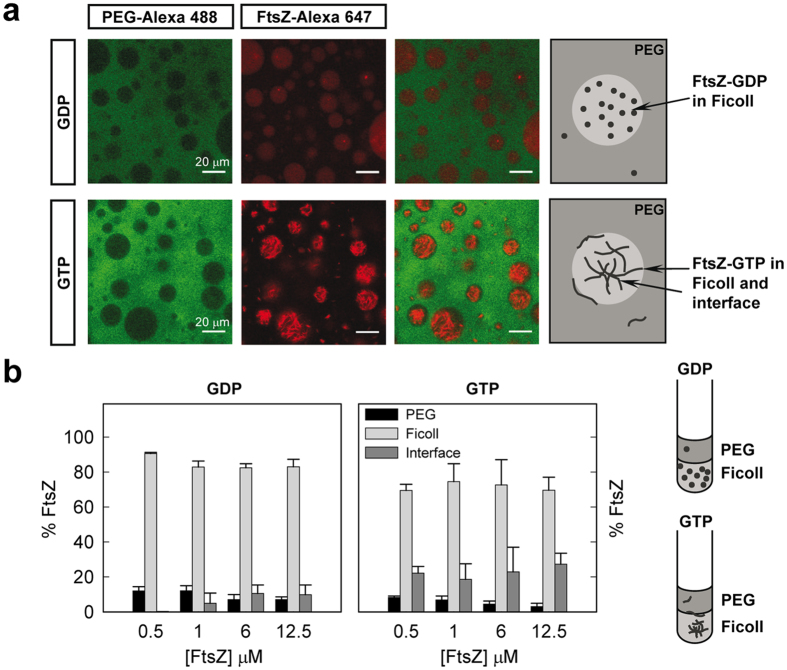
Distribution of FtsZ in the PEG/Ficoll 70 LLPS system. (**a**) Representative confocal images of FtsZ within the PEG/Ficoll (3:1) emulsions in the absence and presence of 1 mM GTP. Total FtsZ concentration was 7 μM. A schematic illustration of the disposition of FtsZ within the phases is depicted on the right. (**b**) Concentration dependence of the distribution of FtsZ within the mixture as determined by fluorescence, together with an illustration on the right, in the absence and presence of 1 mM GTP. Data are the average of at least 3 independent measurements ± SD.

We next analysed the partition of FtsZ-GDP and FtsZ-GTP in PEG/Ficoll 70. We found that the exclusion of unassembled FtsZ-GDP from the PEG-rich phase was even stronger in this mixture than in PEG/dextran 500, as shown by the low levels of colocalisation between the fluorescence signal of the FtsZ-Alexa 647 tracer and the emission of PEG 8 labelled with Alexa 488 (Fig. 4a). The preference of the protein for the phase enriched in Ficoll was also evidenced by fluorescence measurements of the protein content, with most of the protein (>80%) located in this phase, and the remaining 10–15% shared between PEG and the interface (Fig. 4b). The amount of protein in the interface was negligible at low protein concentration and increased slightly with the total FtsZ amount, reaching values comparable to those in PEG/dextran 500 at 12 μM FtsZ. The partition coefficient, around 0.1, was considerably lower than for the PEG/dextran mixtures (Fig. 3), indicating a more uneven partition of FtsZ in the PEG/Ficoll system.

As observed for the unassembled GDP form, FtsZ-GTP was almost completely excluded from the PEG phase, appearing the polymers in the confocal images randomly distributed within the Ficoll phase and, although far less obviously than in PEG/dextran, some of them could also be found at (or close to) the interface with PEG (Fig. 4a and Supplementary Fig. S3). According to our partition coefficient determinations, among both phases FtsZ-GTP stays preferentially in the Ficoll-rich phase (Fig. 4b). Similarly to that previously observed for PEG/dextran 500, where it is however notably higher, the partition coefficient remained basically unaltered with FtsZ concentration and comparable to that for the GDP-form (Fig. 3). Despite the higher dispersity of the measured values for FtsZ polymers, a certain fraction of the protein, only barely higher than with FtsZ-GDP, seemed to locate at the interface and did not significantly vary within the FtsZ concentration interval measured (0.5–12.5 μM; Fig. 4b). At lower FtsZ-GTP concentrations the distribution was similar to that of FtsZ-GDP (Supplementary Fig. S3), probably because of the lack of polymers, suggesting that the minor fraction of protein detected at the interface, even at 0.5–1 μM, is likely assembled due to an enhancement in the association tendency at high concentration of Ficoll, as previously observed^24^. The variability among samples in the content at the interface, particularly at high local concentrations, considering imaging and quantitative data, likely results from the random distribution of the polymers in Ficoll that enables their location anywhere within the phase, including the interface (Supplementary Fig. S3).

From our results it is clear that the distribution and behaviour of FtsZ was different in the PEG/dextran 500 and PEG/Ficoll mixtures. Despite that in both systems the protein was excluded from the PEG-rich phase, the effect was more marked for the Ficoll LLPS system and location of the protein polymers at the interface was more effective with dextran 500. To determine if the observed differences are related with the remarkably different size of the dextran and Ficoll used (500 *vs* 70 KDa) or from their different nature and shape (randomly coiled glucose polymers *vs* roughly globular sucrose polymers^42^), we studied the distribution of FtsZ species in an LLPS system consisting of PEG and dextran T40, more similar in molecular structure to dextran 500 but closer to Ficoll 70 in size. Confocal images of the emulsion showed preferential partition of unassembled FtsZ-GDP into the dextran T40 phase with some of the protein still remaining in PEG (Supplementary Fig. S4), the partition coefficient, 0.57 ± 0.09 at 12.5 μM FtsZ, being close to that for the dextran 500 system. The similarity with the PEG/dextran 500 mixture was further supported by the confocal images of the FtsZ-GTP species showing accumulation of the polymers at the interface around the dextran T40 droplets, and by fluorescence measurements indicating the presence of a significant fraction of protein at the interface that increased with FtsZ concentration (Supplementary Fig. S4). Noteworthy, the fraction of protein (at the highest concentration) at the interface was smaller in the mixture with dextran T40 than with dextran 500. The partition coefficient of FtsZ-GTP polymers was close to that found in the mixture with dextran 500 and, as in this system, remained relatively insensitive to variations in FtsZ concentration (Supplementary Fig. S4).

Therefore, in all the LLPS systems composed of neutral polymers studied, FtsZ strongly partitioned into the non-PEG phase and the distribution was sensitive to its association state. Besides, the LLPS composition determines the level of exclusion from the PEG phase and the fractional amount of protein at the interface as well as its trend with protein concentration.

### Condensation of FtsZ polymers induced by DNA in PEG/DNA mixtures

**Figure 5:**
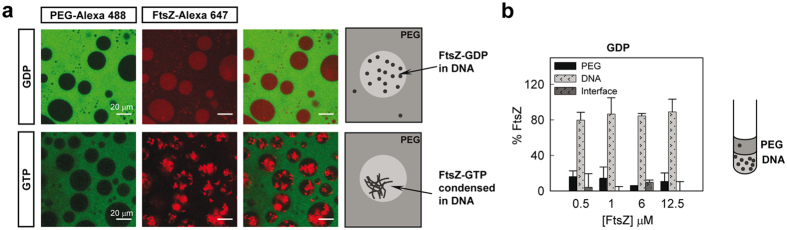
Distribution of FtsZ in the PEG/DNA LLPS system. (**a**) Representative confocal images of FtsZ within the PEG/DNA (3:1) emulsions in the absence and presence of 1 mM GTP. Total FtsZ concentration was 12 (GDP) and 8 μM (GTP). A schematic illustration of the disposition of FtsZ within the phases is depicted on the right. (**b**) Concentration dependence of the distribution of FtsZ-GDP within the mixture as determined by fluorescence together with an illustration on the right. Data are the average of at least 3 independent measurements ± SD.

Nucleic acids, either DNA or RNA, are polymers particularly abundant in the bacterial interior. To address the possible influence of these charged polymers on the partition of FtsZ species, we generated an LLPS system including DNA as one of the components, the other one being PEG. For this purpose we used short DNA fragments prepared according to a previously published protocol^43^, reaching DNA concentrations high enough to achieve phase separation when mixed with PEG. Although this DNA lacks proteins and is consequently less compact than in the bacterial nucleoid, it nonetheless allows us to introduce some of the electrostatic properties of nucleic acid-rich compartments that were absent in the PEG/dextran and PEG/Ficoll systems. Unassembled FtsZ was excluded from the PEG-rich phase to a similar extent than in the PEG/Ficoll mixture as evidenced by the lack of colocalization between FtsZ-Alexa 647 and PEG-Alexa 488 (Fig. 5a). Measurements of the protein content in each phase showed FtsZ-GDP excluded from PEG partitioning mostly within the phase enriched in DNA with negligible amount at the interface (Fig. 5b). The partition coefficient for this mixture was similar to that in PEG/Ficoll (Fig. 3) and lower compared with PEG/dextran.

As for the unassembled FtsZ-GDP species, FtsZ-GTP polymers partitioned preferentially to the DNA phase, but instead of being homogeneously distributed they appeared highly condensed, leaving a noticeable part of the DNA phase free of protein as observed in the confocal images (Fig. 5a). Interestingly, the protein polymers seemed to be expelled from the interface between the DNA droplets and PEG. The behaviour of FtsZ in this mixture was then remarkably different from the other mixtures, in which the polymers were distributed in the Ficoll (randomly) or dextran (evenly) phase and accumulated at the interface to a higher or lesser extent depending on the particular mixture. Fluorescence measurements of the protein content confirmed the large exclusion of the FtsZ polymers from PEG (Supplementary Fig. S5). The anisotropic distribution of the polymers within the DNA made unreliable the measurement of the fraction of protein in this phase. Based on the confocal images, we may assume that no protein locates at the interface and, hence, the amount of protein in the DNA may be, for this particular case, calculated by difference with that determined in PEG. The partition coefficient obtained in this way was close to that for FtsZ-GDP species and that determined in PEG/Ficoll, and remained basically unaltered within the FtsZ concentration interval tested (Fig. 3).

Hence, in the PEG/DNA LLPS system FtsZ was preferentially located in the DNA phase, being homogeneously distributed when unassembled and forming condensed polymers completely excluded from the liquid/liquid interface when polymerization was triggered with GTP.

### Distribution of FtsZ in LLPS systems encapsulated in droplets stabilized by a lipid layer as cell like containers

To better mimic the conditions found in a cell and also study the possible influence of the presence of a lipid boundary in the partition of FtsZ, we developed a procedure to encapsulate LLPS mixtures within a cell-like container surrounded by the ternary mixture of *E. coli* lipids. In this procedure, lipids are dissolved in mineral oil at high concentration and, when in contact with the emulsion of the two phases previously mixed with the protein, they spontaneously encapsulate the mixtures inside water in oil droplets (Supplementary Fig. S6). The stabilization of droplets containing PEG and dextran 500 through the acquisition of a surrounding lipid layer was verified by confocal imaging using a tracer amount of a fluorescently labelled lipid in the ternary mixture and dextran-FITC (Supplementary Fig. S6). With the protocol optimized here we have achieved a high yield of encapsulation of all the LLPS systems studied, PEG/dextran 500, PEG/Ficoll and PEG/DNA using 1:1 to 3:1 volume ratios of the phases, inside multiple droplets of different sizes. Indeed, we found that most of the droplets contained two phases, and sometimes even three phases (two of them equal) with this newly developed procedure.

**Figure 6:**
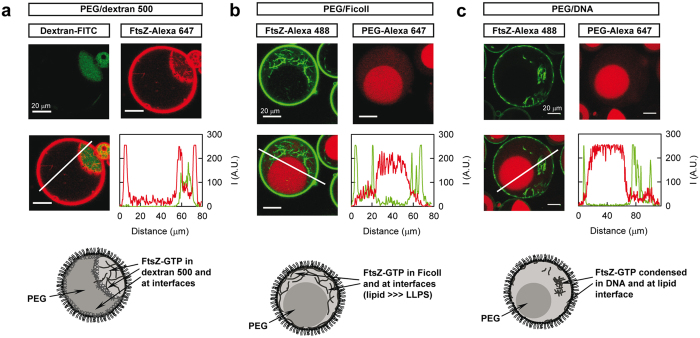
Distribution of FtsZ polymers in several binary mixtures encircled by a lipid layer. (**a**) PEG/dextran 500 (3:1), total FtsZ concentration 7 μM. (**b**) PEG/Ficoll (1:1), total FtsZ concentration 6 μM. (**c**) PEG/DNA (1:1), total FtsZ concentration 12 μM. In all the images of the superimposed channels, lines depict the region through which the intensity profiles for each individual channel (red curve for Alexa 647, green curve for FITC or Alexa 488) were obtained. 1 mM GTP. Cartoons represent the distribution of FtsZ within the encircled phases.

The distribution of FtsZ species inside lipid stabilized droplets containing the different mixtures was analysed by confocal microscopy (Fig. 6 and Supplementary Fig. S7). According to the images, in the PEG/dextran 500 system FtsZ appeared preferentially in the dextran and to a lesser extent in PEG and, when polymerized with GTP, also at the liquid/liquid interface (Fig. 6a and Supplementary Fig. S7a), as observed with the non-encapsulated LLPS system. The colocalisation of FtsZ polymers with dextran-FITC and the accumulation of protein at the interface were clearly observed in the intensity profiles obtained from the corresponding images. A significant amount of FtsZ appeared also at the *E. coli* lipid interface, in good agreement with that previously observed in droplets containing Ficoll^44^ and with unspecific binding measurements using *E. coli* lipid coated microbeads.

Binding to lipids was maintained also with encapsulated PEG/Ficoll 70 mixtures (Fig. 6b and Supplementary Fig. S7b). As in the emulsions without lipids, the presence of the FtsZ polymers at the liquid/liquid interface was reduced regarding that with PEG/dextran and varied from sample to sample with the conditions. When in 1:1 mixtures, FtsZ-GTP located within the Ficoll 70 phase similarly to what was observed in bulk emulsions, and it also randomly appeared at the liquid/liquid interface confirming that there is no particular preference for this region (Fig. 6b). The decrease in the volume of the Ficoll phase in 3:1 mixtures fairly reduced the volume available for FtsZ (as PEG largely excluded FtsZ in this LLPS). Because of the huge increase of the local concentration of FtsZ, bundles arranged to adopt conformations allowing their accommodation within the Ficoll, including alignment with the interface (Supplementary Fig. S3e). This behaviour goes in the line of that previously described in Ficoll containing droplets, where a depletion zone devoid of FtsZ is observed in the vicinity of the lipid layer. This zone, likely formed by the exclusion of stiff polymers from interfacial areas, practically disappeared as got populated with polymers upon increasing protein concentration^44^. In the droplets with PEG and DNA the distribution of FtsZ polymers was, again, significantly different than in the other two systems, with the polymers clustered in the DNA phase leaving most of the volume of this phase and the liquid/liquid interface completely free of protein (Fig. 6c and Supplementary Fig. S7c).

**Figure 7:**
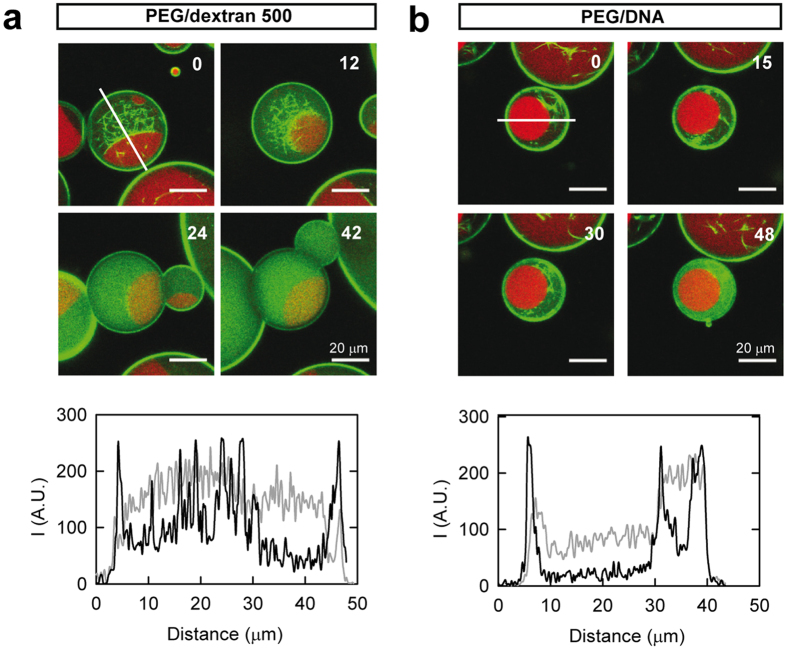
Dynamic behaviour of FtsZ polymers within LLPS systems inside lipid containers. (**a**) PEG/dextran 500 (3:1), total FtsZ concentration 6 μM. Monitoring of depolymerization started 14 min after GTP addition. (**b**) PEG/DNA (1:1), total FtsZ concentration 12 μM. Monitoring of depolymerization started ∼2 h 20 min after GTP addition. In all panels, distribution of FtsZ at the specified time in minutes, time zero being the beginning of visualisation. 0.75 mM GTP. Fluorescence signals correspond to PEG-Alexa 647 (red) and FtsZ-Alexa 488 (green). Lines indicate the region, adapted in each image depending on the orientation of the droplet, through which the intensity profiles below depicting the distribution of FtsZ were obtained (black and grey, zero and end times, respectively). Random variations of the profiles due to subtle movements on the droplet during visualisation were corrected by matching maxima corresponding to the lipid boundary along the x axis.

The assembly dependent location of FtsZ observed in the LLPS mixtures, particularly evident in the PEG/dextran 500 system with or without lipids, suggests that the protein may dynamically relocate from one compartment to another in response to changes in its association state. To prove this hypothesis, we sequentially imaged FtsZ polymers inside droplets stabilized by lipids containing the PEG/dextran 500 mixture (Fig. 7a). Image acquisition started around 15 minutes after GTP addition, time point at which the protein polymers were apparent in the sample, with the expected distribution principally in the dextran-rich phase and at the liquid/liquid interface. In the sequence of images acquired, we observed that the bundles progressively disappeared due to GTP depletion as a result of its hydrolysis by FtsZ, with the concomitant increase of diffuse green fluorescence corresponding to the disassembled protein in the droplet lumen. Interestingly, upon disassembly the protein moved away from the liquid/liquid interface to the phases and preferentially to the dextran 500 (Fig. 7a and Supplementary Movie S1). The lack of polymers in the whole vesicle and not just in the observation plane was demonstrated through a z-scan, and depolymerization was consistently found within all vesicles of the sample after 1 hour (Supplementary Movie S2). We concluded, therefore, that the protein moved from one location to another in response to changes in its association state.

We then took advantage of the stability in time of the LLPS encapsulated in lipid droplets to determine if the condensed FtsZ polymers observed in the PEG/DNA mixture were still dynamic. Image acquisition of these polymers was started around 2 h and 15 minutes after GTP addition, with polymer content similar to that in a sample visualised around 15 minutes after nucleotide addition and the expected distribution within the phases (Supplementary Fig. S8). An increase in the diffuse fluorescence in the green channel indicative of depolymerization was clearly observed with time, mainly within the DNA phase (Fig. 7b and Supplementary Movie S3). In this case, however, depolymerization took place over a remarkably longer time than in the PEG/dextran mixture, the time to achieve full depolymerization was very variable and, apparently, determined by droplet size (Supplementary Movie S4). These results revealed that FtsZ polymers, although condensed in the DNA phase, remain dynamic and retain the ability to hydrolyse GTP.

## Discussion

**Figure 8:**
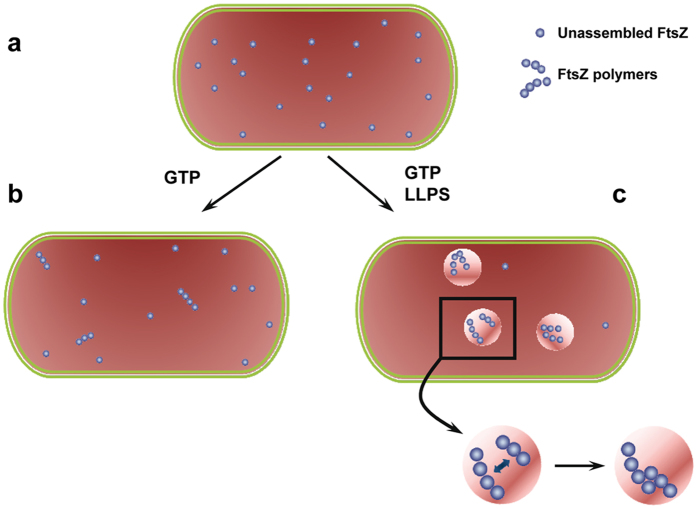
Scheme of the behaviour of FtsZ in LLPS systems. (**a**) In homogeneous media, the FtsZ monomers and small oligomers formed in the presence of GDP would, in principle, distribute randomly. (**b**) GTP triggers the polymerization of FtsZ molecules above the critical concentration. Thus, in these homogeneous media, at low total FtsZ concentration, the protein would largely remain unassembled, even in the presence of GTP. (**c**) Homogeneous crowding reduces the critical concentration of assembly and favours lateral interactions between the filaments. Changes in the local concentration of background molecules may produce phase transitions, and FtsZ molecules would likely distribute preferentially in certain microenvironments, being excluded from others. Under these circumstances, the local increase of FtsZ concentration would further favour its polymerization (overcoming the critical concentration threshold) and bundling. The accumulation of polymers in certain areas may also act as a polymer reservoir under non-division conditions. Besides, this asymmetric distribution would deeply impact the differential molecular recognition of FtsZ modulators and hence FtsZ function.

We have designed here simple LLPS systems to explore the consequences of coexisting membraneless microcompartments on the spatial organization of the essential bacterial division FtsZ protein. We show that FtsZ distributes distinctly in the LLPS systems used to mimic the intracellular heterogeneity and that this distribution is dynamic, largely dictated by the composition of the system, and reversibly modulated by the association state of the protein (Fig. 8). This uneven partition of FtsZ suggests that phase transitions may contribute to the spatial regulation of FtsZ assembly, crucial for its role in division, as local accumulation of the protein would facilitate overcoming the concentration threshold for assembly, while exclusion from other regions would result in disassembly at those regions if the concentration falls below the critical value. In this sense, the accumulation of polymers at certain microenvironments in non-dividing cells could hypothetically serve as a reservoir of soluble functional polymer ready to assemble into a ring when required. The preferential distribution of FtsZ would also have implications for the differential molecular recognition of other biomolecules, favouring the complexes with species/elements accumulated within the same regions over those involving molecules excluded from them. Indeed, there are evidences of FtsZ persisting as patches upon Z-ring disassembly that may act as precursors for its reassembly, also suggested to be involved in the formation of mobile complexes by recruitment of other binding partners^45^. It can be reasonably expected, therefore, that the joint action of phase transitions and assembly/disassembly triggering signals deeply influence the reactivity of FtsZ.

Bacterial division is a process tightly regulated in space and time. The accurate division of a bacterium into two cells of equivalent size and identical genomic material requires the formation of a division ring attached to the membrane and precisely located in the middle of the cell. Bacteria have developed a number of well-known mechanisms for this purpose, most of which operate through the positive or negative regulation of FtsZ polymerization at certain locations^13^,^46^. Recent studies show that, in addition to the mechanisms so far identified, additional factors might contribute to site selection that remain to be determined^20^. Our results suggest that the formation of membrane-free microcompartments as a result of crowding-induced phase transitions may be among those unknown factors affecting the positioning and assembly of the division machinery through modulation of the localisation and distribution of assembled and unassembled FtsZ species.

The behaviour in PEG/DNA is remarkably different from that in the other LLPS studied, being the FtsZ polymers repelled from the interface and their distribution in the DNA phase largely anisotropic. This is probably due to the charged and non-inert nature of DNA, and maybe to its preferential hydration, meaning that it interacts with water molecules stronger than with cosolutes, providing a driving force for their exclusion^47^,^48^. Localisation of the condensed FtsZ bundles in the DNA-rich phase may seem, in principle, unexpected since the divisome assembles at nucleoid free regions to divide the cell without severing the chromosome. However, it has been shown that, under certain conditions, Z-ring assembly can initiate over the centre of the nucleoid^20^, indicating that the nucleoid and FtsZ polymers can share the same spatial region or, at least, are not mutually repelled. Indeed, we have previously shown that the polymerization of FtsZ is dramatically increased in the presence of DNA, likely due to the electrostatic repulsion between both species derived from their negative net charges^24^. Although condensed in the DNA-rich phase, depolymerization with time proves FtsZ polymers remain dynamic, indicating they retain the capability of hydrolysing GTP. The formation of condensed assemblies instead of discrete arrangements of polymers in the DNA phase may prevent formation of a functional constriction ring to protect the chromosome from scission, while the protein remains active to polymerize into a proper ring in locations devoid of DNA. The behaviour of FtsZ polymers found here may be different from that in the areas occupied by the nucleoid due to the different size, structure and compactness of the DNA used compared to the chromosome. In addition, the nucleoid occlusion protein SlmA and other DNA binding proteins may interfere with the formation of condensed FtsZ polymers in the DNA microdomains.

An important fraction of the FtsZ polymers tend to localise at the liquid/liquid interface in the mixtures composed of dextrans, particularly with dextran 500. Interfacial location of large particles has been previously demonstrated^25^,^49^ and, although the mere exclusion of particles from the phases can, at least in part, drive them towards the interface, it seems to be favoured because of the concomitant reduction of surface tension at the aqueous/aqueous interface^41^. In contrast to the PEG/dextran mixtures, FtsZ polymers do not have a particular preference for the interface in the LLPS containing Ficoll and they even seem to be expelled from the PEG/DNA interfaces. Along the same line, differences in the fraction of cells and nanoparticles at the LLPS interfaces have been reported, depending on the specific properties of the particle surface and the type and concentration of the crowders forming the phases system (as composition also impacts interfacial tension)^40^,^50^. In the case of FtsZ polymers, we may speculate that the final distribution is also possibly related with the partition of FtsZ into the two phases in each of the LLPS. Thus, although FtsZ is generally excluded from the PEG phase, it distributes more evenly in the PEG/dextran mixtures compared to the Ficoll or DNA LLPS in which most of the protein is located in the non-PEG phase. The larger repulsion from the dextran phase may further favour its larger location at the interface whereas the preference of FtsZ molecules for the Ficoll or DNA phases may disfavour their interfacial positioning. Localisation of FtsZ polymers at the interface may serve to concentrate them within a defined region, which might enhance or hinder interactions with other division elements. Interfacial assembly favours arrangement of the FtsZ polymers in two rather than three dimensions, which might render a relative orientation more appropriate for constriction.

Lipid encapsulated LLPS systems provide a better platform to evaluate the impact of microenvironments on protein function compared with two-phase emulsions, since the lipid layer limits the size of the systems, bringing them closer to the cellular scale. It also maintains a stable LLPS composition, allowing the study of confinement effects and facilitating the tracking of time-dependent processes. The simultaneous encapsulation of two phases within a lipid container is experimentally challenging though, only attained so far using giant unilamellar vesicles (GUVs) and a single phase formed by two polymers where separation was afterwards induced by physical methods^51^,^52^,^53^. The approach we propose here allows overcoming some of the difficulties associated to these procedures, including the low yield of encapsulation, the limited amount of vesicles, the restriction to certain compositions, and the need for changes in conditions to separate the phases after encapsulation that may be incompatible when including biomolecules. Encapsulation of phases within water in oil droplets has been previously achieved using surfactants to stabilize them^54^ but, to the best of our knowledge, not with lipids as the boundary material, that have been however used for single phase encapsulation in droplets^44^,^55^. Although our model system is not exactly the same as a living bacterial cell, it captures many of its key features as it contains crowding, compartments, microscale volume and a lipid boundary.

Using LLPS systems with controllable composition we have shown that microenvironments driven by phase transitions dynamically modulate FtsZ assembly and localisation. The preference of FtsZ for some compartments may enhance the local concentration of the protein favouring FtsZ assembly at certain locations but not in others from which the protein is excluded. Clearly, the asymmetric distribution of FtsZ between phases can determine its interactions with other molecules involved in division depending on their particular distribution. As phase transitions can occur locally and in a reversible manner, we propose that they may contribute to the fine tuning of bacterial division, likely acting jointly with other specific mechanisms designed for the control of this essential process.

## Methods

### Reagents

Ficoll 70 was from GE healthcare and Dextran 500, PEG 8, GTP nucleotide and other analytical grade chemicals were from Sigma. FITC labelled dextran 500 (dextran 500-FITC) was from Molecular Probes/Invitrogen. Polar extract phospholipids from *E. coli* and lissamine-rhodamine B labelled PE (1,2-dioleoyl-*sn*-glycero-3-phosphoethanolamine-N-(lissamine rhodamine B sulfonyl)), from Avanti Polar Lipids (Alabama, USA), were stored in chloroform at −20 °C.

### Protein purification

*E. coli* FtsZ was purified as described^16^ and stored at −80 °C until use.

### FtsZ and PEG labelling

Labelling of FtsZ with Alexa 488 or Alexa 647 carboxylic acid succinimidyl ester dyes (Molecular Probes/Invitrogen) was performed as described^22^,^56^. Labelling of PEG 8 with the same dyes was conducted by covalently linking them to an amino derivative of the polymer (NH_2_-PEG-NH_2_ from Nanocs). The degree of labelling of FtsZ was estimated from the molar absorption coefficients of the fluorophore and the protein, resulting in 0.5–0.9 moles of fluorophore per mole of FtsZ. The amount of labelled PEG was calculated from the molar absorption coefficient of the corresponding dye.

### DNA fragmentation and purification

DNA (from salmon sperm, Wako Pure Chemical Industries, Osaka, Japan) was subjected to 15 minutes sonication in a bath and purified by the phenol:chloroform:isoamyl alcohol extraction method followed by ethanol precipitation, basically as described^43^. The dried pellet was resuspended in 50 mM Tris-HCl, 300 mM KCl, pH 7.5, and stored at −20 °C until used. Obtained DNA consisted on fragments of up to 300 bp as characterized in agarose gels. DNA concentration was estimated from its dry weight after purification.

### Preparation of LLPS systems

Among the different combinations of the crowders tested, PEG 8, dextrans T40 and 500, Ficoll 70 and DNA, under our experimental conditions (nearly neutral pH, 300 mM KCl and room temperature) and at concentrations compatible with those of our stock solutions, phase separation was only achieved when one of the phases in the mixture was PEG (i.e. with PEG/dextran 500, PEG/dextran T40, PEG/Ficoll 70 and PEG/DNA). This was verified by visual inspection of the tubes and from confocal microscopy images of the fluorescently labelled enriched components after emulsification. Except for DNA (see above) the crowders, without further purification, were equilibrated by extensive dialysis in 50 mM Tris-HCl, 300 mM KCl, pH 7.5. Before use, individual enriched phases were purified as follows. For each particular binary mixture, defined volumes of each crowder were thoroughly mixed to yield a final nominal concentration rendering phase separation as visually determined. These concentrations were 53 and 82 g/L for PEG 8 and dextran 500, respectively; 53 and 82–92 g/L for PEG 8 and dextran T40, respectively; 113 and 165 g/L for PEG 8 and Ficoll 70, respectively; and 92 and 183 g/L for PEG 8 and DNA, respectively. The mixture was centrifuged at 3000 rpm for 5 min in a bench centrifuge to favour phase separation. The stability with time of the system thus separated was checked and no change was found for at least 4 hours. Enriched phases were then isolated by pipetting, discarding the material nearby the interface, and their final concentrations were determined from the refractive index increment (dextran 500 and PEG 8, 0.142 and 0.136 mL/g respectively^57^; dextran T40, 0.147 mL/g (APS Corp); Ficoll 70, 0.141 mL/g^58^). We found that, after equilibration, all the polymers reached a concentration almost twofold their initial concentrations in the mixture, the phase enriched in PEG always lying on top of that with the other polymer in the tubes.

### Preparation of LLPS emulsions

The simplest emulsions were formed by thoroughly mixing the corresponding enriched phases at the desired volume ratio. The mixture PEG/dextran 500 was used to check the distribution of several proportions of the crowders, usually rendering droplets of the minor phase surrounded by the major phase (Supplementary Fig. S9). In general, the 3:1 ratio resulted suitable in all the mixtures for visualisation in bulk. FtsZ, containing a tracer amount of FtsZ-Alexa 488 or FtsZ-Alexa 647 (1 μM), was directly added to this mixture and, when required, polymerization was triggered by diffusion of GTP directly added over the mixture of the two phases containing FtsZ. Emulsions were prepared in working buffer (50 mM Tris-HCl, 300 mM KCl, 1 mM MgCl_2_, pH 7.5). To assess colocalisation of FtsZ with either phase, one of them was labelled with a dye (1 μM final concentration) spectrally different from that of the protein. Images were acquired with different combinations of dyes (FtsZ-Alexa 488 with PEG-Alexa 647, FtsZ-Alexa 647 with PEG-Alexa 488 or dextran-FITC) with equivalent results (Supplementary Fig. S10).

### Preparation of LLPS systems encircled by a lipid membrane

Emulsions were obtained following a procedure based on that described^44^ that we specifically optimized for the encapsulation of binary mixtures. The protocol involves the use of an aqueous and an oil solution. The latter was prepared drying under nitrogen flow, shortly before use, a determined amount of *E. coli* lipids (1.5 mg per sample), subsequently kept under vacuum for at least one hour and dispersing them in mineral oil (Sigma; final concentration 25 g/L) by two cycles of 10 min in a sonication bath. This concentration of lipids in mineral oil was found suitable for the encapsulation of the mixtures. For the aqueous solution, the enriched phases were thoroughly mixed by vortexing to enhance the formation of very small droplets of one in the other. 3:1 or 1:1 volume ratios of PEG to the other phase were employed with similar results. Protein was added on the phases thus mixed and, when required, GTP was also included to a final concentration of 1–2 mM. This mixture was subsequently added on the oil solution containing the dispersed lipids and all components gently mixed by pipetting up and down to render water in oil emulsion droplets of the LLPS systems with protein in working buffer stabilized by lipids (Supplementary Fig. S6).

### Confocal microscopy measurements and data analysis

Emulsions (∼70 μL) were placed in silicone chambers (Molecular probes/Invitrogen) glued to coverslips and visualised by confocal microscopy. Images were collected with a Leica TCS-SP2-AOBS inverted confocal microscope with a HCX PL APO 63x oil immersion objective (N.A. = 1.4–1.6; Leica, Mannheim, Germany). Ar (488–514 nm) and He-Ne (633 nm) ion lasers were used to excite Alexa 488 or FITC and Alexa 647, respectively. DiodeP (561 nm) ion laser was used to excite lissamine-rhodamine. To follow depolymerization of FtsZ inside lipid droplets, a time series was taken every three minutes. Intensity profiles were generated with Image J (National Institutes of Health, USA) within the line defined in the images using the straight line tool.

### Calculation of partition coefficients

Partition coefficients, *K*, within a binary mixture were calculated as the ratio of the concentration of FtsZ in the PEG-rich phase to that in the other phase from evaluation of the fluorescence emission arising from labelled FtsZ using PolarStar Galaxy (BMG Labtech, GmbH, Germany) or Varioskan (Thermo) Plate Readers. FtsZ-Alexa 488 (0.5 μM) and unlabelled FtsZ up to the concentration required in each sample were gently mixed with the phases obtained by mixing the two crowders in working buffer, unless otherwise specified, in a 1:1 volume ratio and allowed to equilibrate until reaching phase separation for 30 min. When required, 1 mM GTP was added to the samples. After centrifugation, phases were isolated (top phase being always PEG) and the fluorescence intensity of an aliquot of each of the phases was measured. The concentration of protein was calculated by comparison with samples containing known amounts of FtsZ-Alexa 488 diluted in the corresponding phase, the signal of which is linear with FtsZ concentration as verified through control measurements. The fraction of FtsZ at the interface was calculated as the difference between the total amount and the sum of those in each phase, except in the case of FtsZ-GTP in the PEG/DNA LLPS system, as explained in the text. Reported values correspond to the average of, at least, 3 independent measurements ± SD.

## Additional Information

**How to cite this article**: Monterroso, B. *et al*. Microenvironments created by liquid-liquid phase transition control the dynamic distribution of bacterial division FtsZ protein. *Sci. Rep.* **6**, 35140; doi: 10.1038/srep35140 (2016).

## Supplementary Material

Supplementary Information

Supplementary Movie S1

Supplementary Movie S2

Supplementary Movie S3

Supplementary Movie S4
