# Supplementary material for: Microenvironments created by liquid-liquid phase transition control the dynamic distribution of bacterial division FtsZ protein
Source: Sci Rep. 2016 Oct 11;6:35140. doi: 10.1038/srep35140 (PMC5057132; doi:10.1038/srep35140)
Supplement: Supplementary Information [file srep35140-s1.pdf]

Supplementary Information

Microenvironments created by liquid-liquid phase transition control the dynamic distribution of bacterial division FtsZ protein

Begoña Monterroso,[1,3]* Silvia Zorrilla,[1,3]* Marta Sobrinos-Sanguino,[1] Christine D. Keating,[2] and Germán Rivas[1]*

[1]Centro de Investigaciones Biológicas, Consejo Superior de Investigaciones Científicas (CSIC), 28040, Madrid, Spain
[2]Department of Chemistry, Pennsylvania State University, University Park, Pennsylvania 16802, USA
[3]These authors contributed equally to this work.

* Corresponding authors: G. R. (grivas@cib.csic.es), S. Z. (silvia@cib.csic.es), B. M. (monterroso@cib.csic.es). Centro de Investigaciones Biológicas, Consejo Superior de Investigaciones Científicas (CSIC). Ramiro de Maeztu, 9. 28040, Madrid, Spain. Tel. +34 918373112.

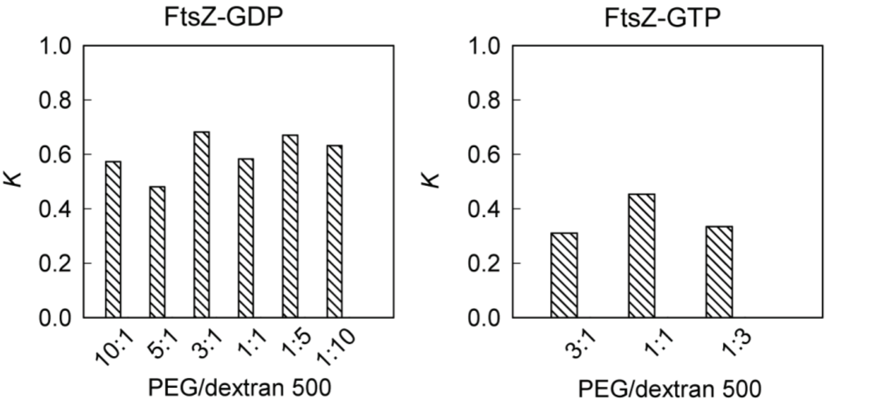

Supplementary Figure S1. Evolution of the partition coefficient K of FtsZ species. Partition coefficients of FtsZ-GDP (left) and FtsZ-GTP (right) as a function of the volume ratios in PEG/dextran 500 LLPS. FtsZ concentrations were 1 and 12.5 µM, respectively.

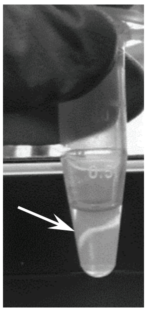

Supplementary Figure S2. Distribution of FtsZ polymers within the PEG/dextran 500 LLPS. Both phases were mixed in a 1:1 ratio. PEG locates at the top, dextran at the bottom. FtsZ concentration was 12.5 µM. The accumulation of protein at the interface is indicated with an arrow.

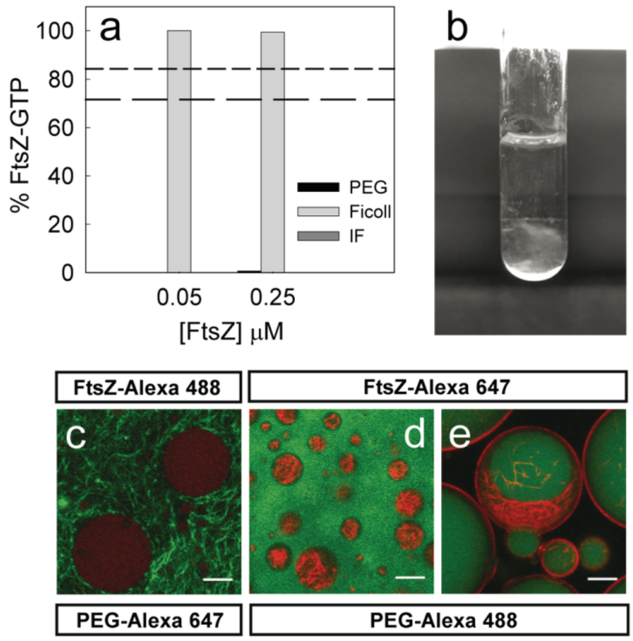

Supplementary Figure S3. Distribution of FtsZ-GTP in the PEG/Ficoll 70 LLPS. (**a**) Partition of FtsZ-GTP at low protein concentrations. IF, interface. For reference, the average content measured in the Ficoll phase of this LLPS for FtsZ-GDP (short-dashed line) and FtsZ-GTP at higher FtsZ concentrations (at and above 0.5 µM; long-dashed line) are depicted. (**b**) Distribution of 12.5 µM FtsZ-GTP. PEG locates at the top, Ficoll at the bottom. (**c**) and (**d**) PEG/Ficoll (3:1) in bulk emulsion, total FtsZ concentration was 9 and 7 µM, respectively. (**e**) PEG/Ficoll (3:1) inside lipid droplets, total FtsZ concentration was 8 µM. Bars are 20 µm.

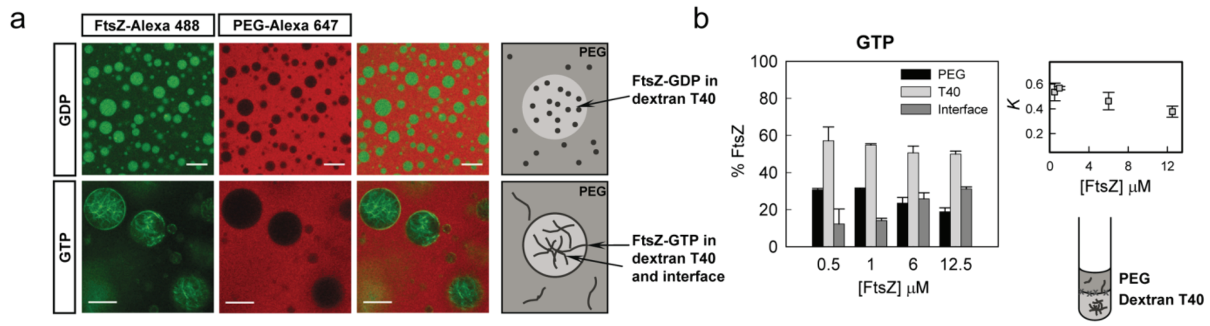

Supplementary Figure S4. Distribution of FtsZ in the PEG/dextran T40 LLPS. (**a**) Representative confocal images of FtsZ within the PEG/dextran T40 (3:1) LLPS in the absence and presence of 1 mM GTP. Total FtsZ concentration was 8 µM. Bars are 20 µm. A schematic illustration of the disposition of FtsZ within the phases is depicted on the right. (**b**) Concentration dependence of the percentage of FtsZ-GTP within the LLPS as determined by fluorescence. Evolution of the partition coefficient *K* with FtsZ concentration is shown on the right, together with a schematic illustration of the disposition of FtsZ polymers within the phases.

Supplementary Figure S5. Concentration dependent distribution of FtsZ polymers in the PEG/DNA LLPS. The concentration of FtsZ in the PEG phase was measured by fluorescence, and that in DNA was assumed to be the difference with the total amount of protein.

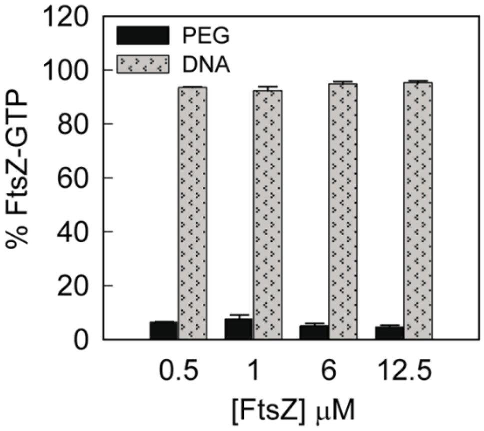

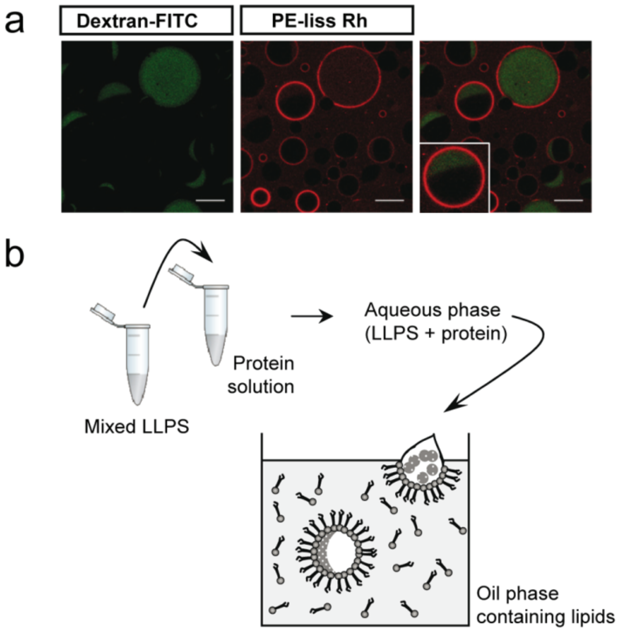

Supplementary Figure S6. Encapsulation method of LLPS into lipid stabilized droplets. (**a**) Representative images of the LLPS encapsulated into lipid containers. *E. coli* lipids were doped with the rhodamine labelled phosphatidylethanolamine and used to encapsulate the PEG/dextran 500 (3:1) LLPS. Inset displays a magnification evidencing the presence of both phases inside the lipid container. Bars are 40 µm. (**b**) Scheme of the procedure followed to encapsulate LLPS into containers stabilized by a lipid layer.

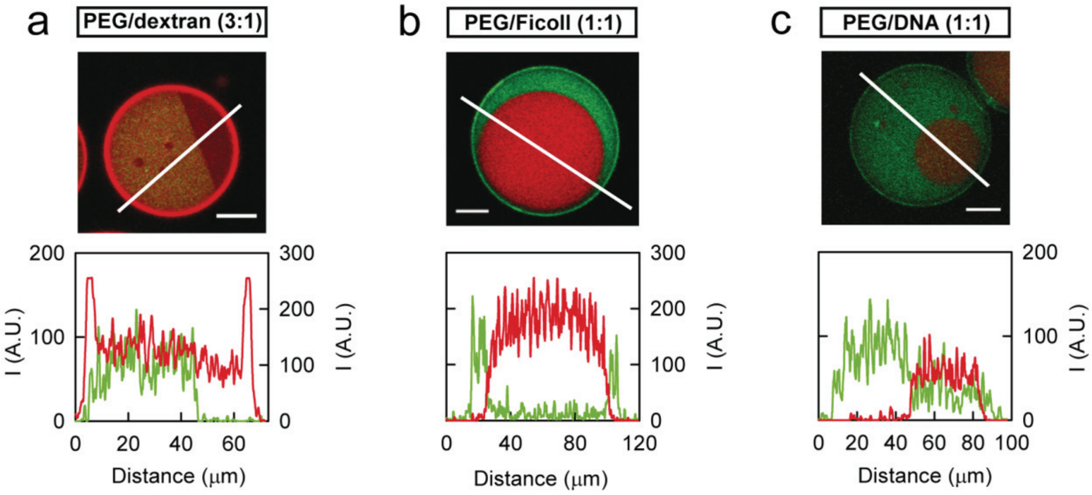

Supplementary Figure S7. Distribution of FtsZ-GDP in LLPS encircled by a lipid layer. (**a**) Fluorescence signals correspond to dextran 500-FITC (green) and FtsZ-Alexa 647 (red). Total FtsZ concentration was 7 µM. (**b**) and (**c**) Fluorescence signals correspond to PEG-Alexa 647 (red) and FtsZ-Alexa 488 (green). Total FtsZ concentration was 6 and 12 µM, respectively. In all the images, lines depict the region through which the intensity profiles for each individual channel (red curve for Alexa 647, green curve for FITC or Alexa 488) were obtained. Bars are 20 µm.

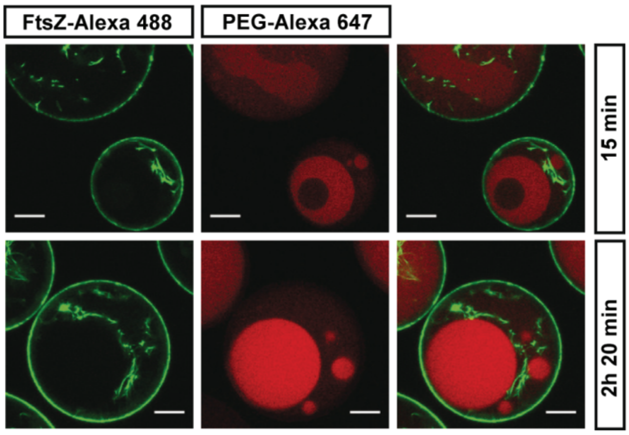

Supplementary Figure S8. Distribution with time of FtsZ-GTP within PEG/DNA (1:1) LLPS encapsulated by a lipid layer. Total FtsZ concentration was 12 µM. Images were taken at the specified times after GTP addition (0.7 mM). Superimposition of both channels is depicted on the right. Bars are 20 µm.

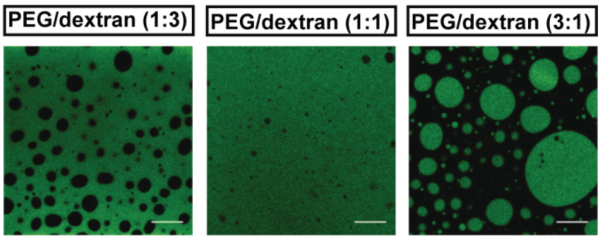

Supplementary Figure S9. Distribution of the PEG/dextran 500 LLPS emulsions at varying v/v ratios. Fluorescence signal corresponds to dextran 500-FITC, used as a tracer for the dextran 500 phase. Bars are 40 µm.

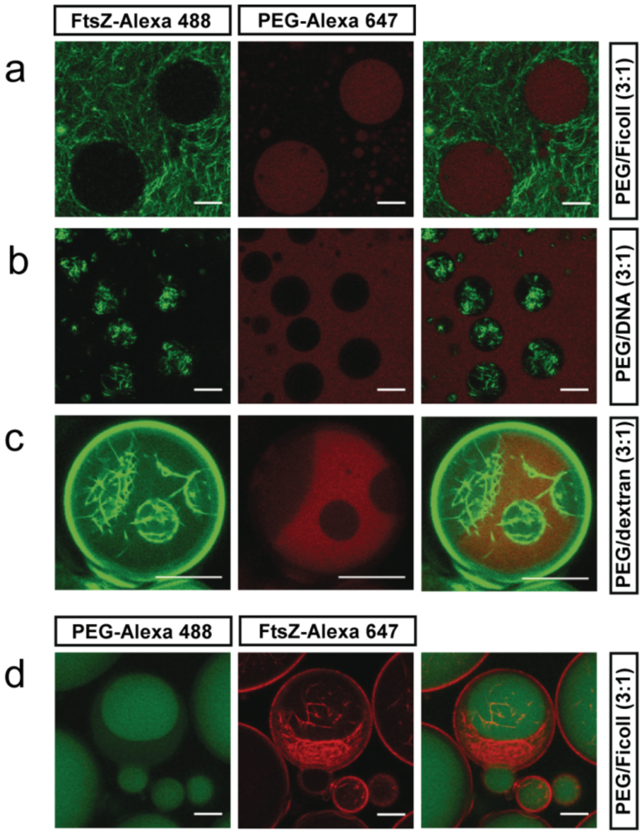

Supplementary Figure S10. Distribution of FtsZ-GTP within LLPS in bulk emulsion or encapsulated by a lipid layer. (**a**) and (**b**) LLPS in bulk emulsion, total FtsZ concentration was 9 µM. (**c**) and (**d**) LLPS inside lipid droplets, total FtsZ concentration was 6 and 8 µM, respectively. Superimposition of both channels is depicted on the right. Bars are 20 µm.

Legends for Supplementary Movies

Supplementary Movie S1. Assembly dependent localisation of FtsZ in encapsulated PEG/dextran 500 LLPS. Evolution of FtsZ polymers inside droplets during 50 min. Time zero corresponds to 15 min after GTP addition.

Supplementary Movie S2. z-scan after FtsZ depolymerization in encapsulated PEG/dextran 500 LLPS. FtsZ depolymerization is consistent in all vesicles within the same sample after 1 h.

Supplementary Movie S3. Assembly dependent localisation of FtsZ in encapsulated PEG/DNA LLPS. Evolution of FtsZ polymers inside droplets during 50 min. Time zero corresponds to 2h 15 min after GTP addition.

Supplementary Movie S4. z-scan after FtsZ depolymerization in encapsulated PEG/DNA LLPS. FtsZ depolymerization is variable among droplets within the same sample after around 3h.
